# Enhanced passive safety surveillance of a quadrivalent inactivated split virion influenza vaccine in Finland during the influenza season 2020/21

**DOI:** 10.1186/s12889-022-13898-z

**Published:** 2022-08-08

**Authors:** Olga Syrkina, Ajinkya Inamdar, Sophie Wague, Céline Monfredo, Markku Nissilä, Anne-Laure Chabanon, Laurence Serradell

**Affiliations:** 1 Sanofi, Swiftwater Campus, 1 Discovery Drive, Swiftwater, PA 18370 USA; 2 Sanofi, Siège Mondial Campus Sanofi Lyon, 14 Espace Henry Vallée, 69007 Lyon, France; 3 Global Biostatistical Sciences, Sanofi, 1541 Avenue Marcel Mérieux, 69280 Marcy l’Etoile, France; 4 Terveystalo Biobank and Clinical Research, Humalistonkatu 7b, 20100 Turku, Finland

**Keywords:** Seasonal influenza, Influenza vaccine, IIV4, Vaxigrip Tetra, Quadrivalent split-virion inactivated influenza vaccine

## Abstract

**Background:**

The European Medicines Agency (EMA) requires enhanced safety surveillance to be conducted for annual seasonal influenza vaccines with the aim of rapidly detecting any potential new safety concerns before the peak immunisation period of the vaccine in any given year. The aim of this study was to detect any clinically significant change in the frequency or severity of expected reactogenicity of the quadrivalent inactivated split-virion influenza vaccine (IIV4) during routine immunisation in Finland for the 2020/21 season. The primary objective was to investigate the frequency of suspected adverse drug reactions (ADRs) occurring within 7 days following vaccination.

**Methods:**

Enhanced passive safety surveillance of individuals vaccinated with IIV4 was conducted from October 9, 2020 to November 30, 2020 across seven sites in Finland. The vaccinee reporting rate and ADR reporting rate were calculated and compared with known or expected safety data in order to identify any clinically significant changes.

**Results:**

Data were collected from 1008 individuals with 29 vaccinees reporting 82 suspected ADRs. Of these, 28 people reported 79 suspected ADRs within 7 days following vaccination, corresponding to a vaccinee reporting rate of 2.78% (95% CI: 1.85, 3.99) (ADR reporting rate, 7.84% [95% CI: 6.25, 9.67%]). The most frequently reported ADRs were injection site reactions (vaccination site pain, vaccination site erythema and vaccination site swelling) (*n* = 46, 2.28%), myalgia (*n* = 9, 0.89%) and headache (*n* = 8, 0.79%). No serious suspected adverse events were reported at any point post-vaccination and ADR reporting rates were in general lower compared to those reported for IIV4 in the 2019/20 surveillance study.

**Conclusion:**

No clinically significant changes in what is known or expected for IIV4 were reported for the 2020/21 season which supports the safety profile of this vaccine and will help maintain public confidence in influenza vaccination.

**Supplementary Information:**

The online version contains supplementary material available at 10.1186/s12889-022-13898-z.

## Background

Seasonal influenza epidemics are estimated to cause between 291,243 and 645,832 deaths annually with an excess mortality rate of 0.1–6.4 per 100,000 individuals for those < 65 years of age and 17.9–223.5 per 100 000 for those older than 75 years [1].

Annual seasonal influenza vaccination is the primary strategy used globally to limit the burden of seasonal influenza [2, 3]. Annual vaccination is recommended by the World Health Organization (WHO) for high-risk groups including pregnant women, children aged < 5 years, the elderly, and individuals with chronic health conditions [4, 5]. However, current influenza vaccines generally induce strain-specific immunity. As influenza virus strains continuously evolve through antigenic drift resulting in new variants or strains [2], vaccines need to be frequently updated to match the strains most likely to be circulating during the upcoming season [2]. This leads to specific challenges for the continued monitoring of vaccine safety.

Since 2014, the European Medicines Agency (EMA) has required manufacturers of seasonal influenza vaccines to conduct annual enhanced safety surveillance, replacing previous requirements based on data from small-scale safety and immunogenicity clinical trials [6, 7]. Specific guidance has been produced by the EMA’s Pharmacovigilance Risk Assessment Committee (PRAC) for enhanced safety surveillance [6]. The purpose of this surveillance is to rapidly detect and evaluate potential new safety concerns before the peak immunisation period of the vaccine in any particular year. Increased rates of reactogenicity or allergic events compared to those expected or reported following vaccination during the the previous season may signal new safety concerns and may indicate a potential for increased severity with increased vaccine exposure [6]. The use of enhanced safety surveillance to monitor adverse drug reactions (ADRs) is recommended by the EMA for the purpose of regulatory submission and review [6]. Using a passive surveillance system based on individual and healthcare professional (HCP) reporting of ADRs has been shown to improve the rates of adverse event (AE) reporting in comparison to previous years where passive surveillance systems relied on ADR reporting by HCPs alone [8, 9].

In 2016, a quadrivalent inactivated split-virion influenza vaccine (IIV4; Vaxigrip Tetra®, Sanofi), which offers broad protection against influenza through the inclusion of two influenza A and two influenza B virus strains, was approved in Europe [10]. IIV4 is indicated for use in adults and children from 6 months of age [11].

Several adjustments have been made to methods of enhanced passive safety surveillance (EPSS) reporting which have been used for for IIV4 surveillance over previous years, with the aim of improving both the quality of the data and the reporting stimulus. The introduction of a combination of a paper reporting method, using a pre-paid envelope, and phone reporting to replace email reporting, during the 2019/20 EPSS for individuals vaccinated with IIV4 provided better quality reporting. This was demonstrated by the higher than expected rates of spontaneous reporting (6.0%) in comparison to the method of email reporting alone that was used during the northern hemisphere 2018/19 EPSS for IIV4 [4, 12].

Here we report an EPSS for individuals vaccinated with IIV4 during the northern hemisphere (NH) 2020/21 season, in which we used electronic reporting via an electronic data capture system (EDC), rather than email or mail, in addition to the standard reporting method by telephone.

The overall aim of this study was to detect any clinically significant change in the frequency or severity of expected reactogenicity, compared with what is known or expected with the vaccine as defined by the Summary of Product Characteristics (SmPC). We also described the estimated reporting rates of suspected ADRs occurring within 7 days following routine vaccination with IIV4 and compared them with previous data for the EPSS 2019/20 influenza season and with expected frequencies as per the SmPC.

## Methods

### Study design, population and setting

Between October 9, 2020 and November 30, 2020, an EPSS was conducted to examine ADRs associated with IIV4 vaccination within seven participating sites (private clinics) in Finland. This EPSS included individuals aged 6 months and older who had received IIV4 from their HCP within 4 to 6 weeks following the start of seasonal influenza vaccination. Vaccines were administered as per routine clinical practice in accordance with the product labelling (indication).

The study was conducted in accordance with the Declaration of Helsinki, Good Epidemiological Practice, and the European Network of Centres for Pharmacoepidemiology and Pharmacovigilance. Informed consent was not required because the EPSS relied on routine pharmacovigilance and voluntary spontaneous reporting.

### Vaccine formulation

The IIV4 vaccine contained 15 μg haemagglutinin per strain of A/Guangdong Maonan/SWL1536/2019 (H1N1) pdm09-like strain (A/Guangdong-Maonan/SWL1536/2019, CNIC-1909), A/Hong Kong/2671/2019 (H3N2)-like strain (A/Hong Kong/2671/2019, IVR-208), B/Washington/02/2019-like strain (B/Washington/02/2019, wild type) and B/Phuket/3073/2013-like strain (B/Phuket/3073/2013, wild type) within a 0.5 mL dose. All strains were propagated in fertilised hens' eggs from healthy chicken flocks [13].

### Endpoints

The primary endpoint was the occurrence of suspected ADRs within 7 days following routine vaccination with IIV4 during the NH influenza season 2020/21. Secondary endpoints included the occurrence of suspected ADRs occurring within 7 days following routine vaccination with IIV4 according to pre-defined age groups (≥ 6 months to < 6 years, ≥ 6 to < 13 years, ≥ 13 to < 18 years, ≥ 18 to ≤ 65 years and > 65 years); the occurrence of serious suspected ADRs after vaccination with IIV4 at any time following vaccination; and vaccinee reporting rates of suspected ADRs observed during the NH influenza season 2020/21 compared with vaccinee reporting rates of suspected ADRs observed during the NH influenza season 2019/20, or compared with the frequencies documented in the SmPC. Serious suspected ADRs were defined as ADRs that result in death or are life threatening, or that require inpatient hospitalization or the prolongation of existing hospitalization, or result in the persistence of significant disability or incapacity.

### Study conduct and data collection

Following vaccination with IIV4, the HCPs informed each vaccinee or vaccinee’s parents/legal guardians on the importance of reporting suspected ADRs, especially those occurring within the first 7 days, and instructed them on how to use the EDC system (Viedoc 4.60), which was set up using vaccinee-specific accounts, to report any ADRs directly in ‘real-time’. Alternatively, vaccinees or vaccinee’s parents/legal guardians could report ADRs by phone to the Finnish Sanofi Medical Information & Pharmacovigilance Call Centre and the ADRs reported to this centre were integrated into the pharmacovigilance database. The ADRs recognized as being of particular interest by the PRAC were analysed separately and classified as PRAC ADRs of interest. The following were defined by the PRAC as ADRs of interest: systemic reactions (fever [≥ 38 °C], nausea, vomiting, malaise, headache, decreased appetite, myalgia and/or arthralgia, irritability/prolonged crying [for children under 5 years of age]), injection-site reactions (pain, erythema and swelling) and events indicative of allergic and hypersensitivity reactions, including rash and ocular symptoms.

Vaccination cards (VC) were provided to the vaccinee or vaccinee’s parents/legal guardians to facilitate data collection. A copy of the VC was retained at the site so that the details could be transcribed into the EDC (including the unique patient identifier automatically generated using the EDC, batch number of vaccine, vaccination date and vaccinee age group). All data were extracted from the PV (pharmacovigilance) database for the statistical analyses of the safety data.

### Statistical analysis

Descriptive statistics were used to summarise the data, including the vaccinee reporting rate and ADR reporting rate, with associated two-sided 95% confidence intervals (CI). The vaccinee reporting rate was calculated using the number of vaccinees who reported at least one suspected ADR divided by the total number of VCs distributed; and the ADR reporting rate was calculated using the number of suspected ADRs divided by the total number of VCs distributed. The ADR reporting rate 95% CIs were calculated using the Clopper-Pearson exact CI while the vaccinee reporting rate 95% CIs were calculated using the normal approximation method of binomial CI (Wald asymptotic CI) or using the Clopper-Pearson exact CI where applicable.

The EPSS current interim guidance [6] for seasonal influenza vaccines in the EU (EMA/PRAC/222346/2014) requires the system to be able to detect ADRs considered to be common, i.e. expected to occur with a reporting rate of ≥ 1%. To meet this requirement, 1000 individuals were targeted to be vaccinated within 4 to 6 weeks following the start of the influenza vaccination season across the participating sites. This population size provides a > 99% probability of collecting ≥ 1 report of a common AE. Data were summarised cumulatively by pre-defined age group, by ADRs occurring ≤ 7 or > 7 days after vaccination or missing, by seriousness and by severity.

The previous reporting rates obtained in the EPSS NH influenza season 2019/20 were used as a comparator for the reporting rates observed in the current EPSS NH influenza season 2020/21 in addition to SmPC, in order to evaluate any potential increase in reactogenicity. Any reports received outside the EPSS period were handled as routine spontaneous reports but were not included in the analysis.

## Results

A total of 1008 participants were included; most (99.8%) were ≥ 18 years old. In total, 29 reports were received for 82 suspected ADRs. The time to ADR onset was known for 79 (79/82; 96.3%) ADRs, all of which occurred within the first 7 days of vaccination. Of these, the majority (70/79; 88.61%) occurred on the same day or the following day after vaccination.

For the primary endpoint, 28 participants who received the IIV4 vaccine reported 79 suspected ADRs within 7 days following vaccination, corresponding to a vaccinee reporting rate of 2.78% (95% CI: 1.85, 3.99) (ADR reporting rate, 7.84% [95% CI: 6.25, 9.67%]) (Table 1). Among older participants (> 65 years), the vaccinee reporting rate was 6.25% (95% CI: 0.77, 20.81), while those aged 18–65 years had a vaccinee reporting rate of 2.67% (95% CI: 1.73, 3.91) (ADR reporting rates of 15.63% [95% CI: 5.28, 32.79] and 7.58% [95% CI: 5.97, 9.46], respectively). No ADRs were reported by those aged 13–18 years (number of VCs distributed, *n* = 4) and of only two participants in the 6–13 years age group, one reported 3 ADRs (Table 1). Seventy-two PRAC ADRs of interest occurred in 27 vaccinees within 7 days following vaccination with IIV4, corresponding to a vaccinee reporting rate of 2.68% (95% CI: 1.77, 3.87%) (PRAC ADR reporting rate: 7.14% [95% CI: 5.63, 8.91%]) (Table 1).

**Table 1:** Adverse drug reaction rates stratified by age, reported within 7 days of vaccination

	**VC distribution (n)**	**Vaccinees reporting ≥ 1 ADR (n)**	**Vaccinees reporting rate (%) (95% CI)**	**ADRs (n)**	**ADR reporting rate (%) (95% CI)**
ADR	1008	28	2.78 (1.85,3.99)	79	7.84 (6.25, 9.67)
PRAC ADRs of interest	1008	27	2.68 (1.77, 3.87)	72	7.14 (5.63, 8.91)
6–13 years of age	2	1	50.00 (1.26, 98.74)	3	150.00 (NE)
13–18 years of age	4	0	0.00 (0.00,60.24)	0	0.00 (0.00, 60.24)
18–65 years of age	937	25	2.67 (1.73, 3.91)	71	7.58 (5.97, 9.46)
> 65 years of age	32	2	6.25 (0.77, 20.81)	5	15.63 (5.28, 32.79)

Vaccinees reporting rate, number of vaccinees who reported ≥1 ADR divided by the number of vaccine cards distributed × 100%; ADR reporting rate, number of ADRs reported divided by the number of vaccine cards distributed × 100%

*ADR* adverse drug reaction, *CI* confidence interval, *NE* not estimated

Reporting rates for all ADRs (including those without known time to onset) did not differ substantially from those known to have occurred within the first 7 days (Table 2). The most frequently reported ADRs were injection site reactions (vaccination site pain, vaccination site erythema and vaccination site swelling; *n* = 46, 2.28%), myalgia (*n* = 9, 0.89%) and headache (*n* = 8, 0.79%) (Table 3). No serious suspected ADRs were reported at any point post-vaccination. However, two ADRs (vaccination site swelling and headache) were of severe intensity; these were both PRAC ADRs of interest.

**Table 2:** Comparison of reporting rates of suspected ADRs between NH influenza Season 2019/20 and NH influenza Season 2020/21

	**NH Influenza Season 2019/2020**^a^		**NH Influenza Season 2020/2021**	
	** ≤ 7 days**	**Total**	** ≤ 7 days**	**Total**^**b**^
**Total number of VCs distributed**		939		1008
**Overall ADRs**				
** Total number of vaccinees who reported at least one suspected ADR**	38	56	28	29
** Vaccinees reporting rate, % (95% CI)**	4.05 (2.79, 5.31)	5.96 (4.45, 7.48)	2.78 (1.85, 3.99)	2.88 (1.94, 4.11)
** Total number of suspected ADRs**	117	163	79	82
** ADR reporting rate,% (95% CI)**	12.46 (10.41, 14.74)	17.36 (14.99, 19.94)	7.84 (6.25, 9.67)	8.13 (6.52, 10.00)
**PRAC ADRs of interest**				
** Total number of vaccinees who reported at least one PRAC ADRs of interest**	36	56	27	28
** Vaccinees reporting rate, % (95% CI)**	3.83 (2.61, 5.06)	5.96 (4.45, 7.48)	2.68 (1.77, 3.87)	2.78 (1.85, 3.99)
** Total number of PRAC ADRs of interest**	77	112	72	74
** PRAC ADRs of interest reporting rate,% (95% CI)**	8.20 (6.53, 10.14)	11.93 (9.92, 14.17)	7.14 (5.63, 8.91)	7.34 (5.81, 9.13)

*ADR* adverse drug reaction, *CI* confidence interval, *PRAC* Pharmacovigilance Risk Assessment Committee (PRAC ADRs of interest were ADRs recognized as being of particular interest by the Pharmacovigilance Risk Assessment Committee)

^a^Data obtained from EPSS conducted during the NH 2019/20 flu season [12]

^b^Includes unknown time to onset

**Table 3:** Vaccinee reporting rate by PRAC adverse drug reactions occurring within 7 days after vaccination

	Total number of VCs distributed *N* = 1008	
	**n**	**(%) (95% CI)**
**PRAC ADRs of interest**	72	7.14 (5.63, 8.91)
**Injection site reactions**	45	2.18 (1.37, 3.29)
Vaccination site pain	19	1.88 (1.14, 2.93)
Vaccination site erythema	15	1.49 (0.84, 2.44)
Vaccination site swelling	11	1.09 (0.55, 1.94)
**Myalgia**	9	0.89 (0.41, 1.69
**Headache**	7	0.69 (0.28, 1.43)
**Fever**	3	0.30 (0.06, 0.87)
**Nausea**	3	0.30 (0.06, 0.87)
**Arthralgia**	2	0.20 (0.02, 0.71)
**Malaise**	2	0.20 (0.02, 0.71)
**Allergic and hypersensitivity reactions**	1	0.10 (0.00, 0.55)

ADR, adverse drug reaction; CI, confidence interval; PRAC, Pharmacovigilance Risk Assessment Committee (PRAC ADRs of interest were ADRs recognized as being of particular interest by the Pharmacovigilance Risk Assessment Committee); VC, vaccination card

The total vaccinee reporting rates and ADR reporting rates tended to be lower in the current season (2020/21) than in the previous season (2019/20), for suspected ADRs occurring ≤ 7 days post vaccination (Table 2).

A number of PRAC ADRs of interest reported in the current EPSS were not observed in the previous year. These included 11 reports of vaccination site swelling reported by 11 vaccinees (vaccinee reporting rate, 1.09% [95% CI: 0.55, 1.94]): 1 in the ≥ 6 to < 13 years age group, 9 in the ≥ 18 to ≤ 65 years age group and 1 aged > 65 years. This was in line with the expected frequency stated in the SmPC, which lists vaccination site swelling as common (1% to 10%) in adults and the elderly and very common (≥ 10%) among children aged 6 to 13 years. The other PRAC ADRs of interest were reported in both previous and current seasons.

## Discussion

In this EPSS conducted in Finland during the NH 2020/21 influenza season, 2.78% of the vaccinees reported 79 suspected ADRs within 7 days following vaccination with IIV4. These findings were favourable compared with those reported in the EPSS from the previous year in Finland (overall vaccinee reporting rate for the previous year (2019/20), 5.96% [95% CI: 4.45, 7.48%] and ADR reporting rate, 17.36% [95% CI: 14.99, 19.94%]) [12]. Furthermore, the safety profile was comparable to that described in the SmPC [13]. For the primary objective, no safety concerns were observed during the surveillance period, in line with findings from EPSS studies conducted in previous influenza seasons [4, 12, 14]. Over half of the events reported during the current EPSS were of mild intensity and 2.5% of ADRs reported were of severe intensity (vaccination site swelling and headache). 

For the current season, we implemented electronic reporting via the EDC using vaccinee-specific accounts in addition to the standard phone reporting method. While this digital reporting strategy provided the expected good quality of reporting, reporting rates were surprisingly lower versus the paper reporting method (pre-paid envelope) used in the 2019/20 season. Possible reasons for this include the inherent risks associated with using a digital solution, such as technical issues in accessing the EDC in time or a lack of vaccinee adherence to the EDC solution, thereby possibly hindering ADR reporting. This is however unlikely as reporting via telephone was provided as a back-up solution. Additionally, only one vaccinee reported facing technical difficulties with the EDC. Overall, the EDC solution was found to be well accepted by the vaccinees, with no negative feedback during follow-ups by the sites/ local PV. The current disruption to health-seeking behaviour and staffing/routines in clinics and to surveillance priorities and capacities as a result of the ongoing coronavirus disease 19 (COVID-19) pandemic has led to a decrease in influenza information sharing globally [15]. Such disruptions during the 2020/21 influenza season could be one explanation for the lower reporting rates observed in this study. It is also possible that confidence in flu vaccines has increased over time, with IIV4 being on the market for at least three seasons. 

The vaccinee reporting rate reported here for IIV4 is in line with published reporting rates of spontaneous ADR reports after vaccination with other seasonal influenza vaccines, ranging from 20 to 90 reports per 1,000,000 people vaccinated [16–20]. The ability of EPSS to improve reporting rates following vaccination has been previously demonstrated in a number of studies [8, 14, 16, 21, 22]. In an Australian investigation, the introduction of EPSS increased the reporting rates for vaccines administered in the state of Victoria from 2.6 reports per 100,000 in 2003 to 13.5 reports per 100,000 people vaccinated per annum in 2009 [21], while in an Italian study reporting rates for the seasonal influenza vaccine increased from 55 reports per 1,000,000 for the period 2009-2012 to approximately 140 reports per 1,000,000 doses in 2014 following the introduction of EPSS [8]. 

The surveillance carried out in this study enabled near real-time reporting of ADRs after vaccine exposure through the collection of data within a short time period at the start of the seasonal influenza vaccination. This was important to mitigate potential risks before the peak period of the influenza season. Spontaneous reporting was stimulated by HCPs informing vaccinees or vaccinees’ parents/legal guardians about the importance of reporting ADRs and was further facilitated using VCs and enabling the vaccinees or the parents/legal guardians to report the ADRs directly using the EDC system. The customised reporting form containing predefined ADRs and focused questions enabled the collection of accurate and complete information for case processing and follow-up by local PV.

In this EPSS, both younger and older age groups were under-represented. The study included individuals from private clinics, whereas influenza vaccines were distributed for free at public health clinics in Finland. Therefore, there may have been a lack of economic incentive for parents to bring their children or for older adults to attend private clinics to receive the vaccine. Although the enrollment was low in these age groups, considering the descriptive nature of the analyses, it is unlikely that this would have significantly impacted the findings of the EPSS.

It should also be noted that all cases were self-reported by vaccinees or their legal guardians and were not medically confirmed. However, all cases were non serious and it is unlikely that they would have triggered a medical visit; thus confirmation by an HCP would not have been obtained for most ADRs reported. As is inherent in passive surveillance, and to avoid bias in the collection of safety data, there was no possibility to follow-up with individuals at the site level. The interpretation of our findings should take into account the possibility of both under-reporting (where only a fraction of the total number of ADRs actually occurring after vaccination are reported) and differential reporting (where ADRs and ADRs with shorter time to onset after vaccination are more likely to be reported during the surveillance period than ADRs with longer time to onset). 

## Conclusions

In conclusion, the 2020/21 EPSS results did not suggest any clinically significant change in what is known or expected in terms of the safety of IIV4. The analysis of suspected ADRs did not identify any reporting pattern by type, frequency or severity, supporting the established acceptable safety profile of this vaccine. These findings will help to maintain public confidence in influenza vaccination.

## Supplementary Information

**Additional file 1: Table S1**. Vaccinee reporting rate by PRAC adverse drug reactions occurring within 7 days after vaccination.

## Data Availability

Qualified researchers may request access to patient-level data and related documents [including, e.g., the clinical study report, study protocol with any amendments, blank case report form, statistical analysis plan, and dataset specifications]. Patient-level data will be anonymized, and study documents will be redacted to protect the privacy of trial participants. Further details on Sanofi’s data sharing criteria, eligible studies, and process for requesting access can be found at: https://www.vivli.org/.

## Abbreviations

ADR: Adverse drug reaction
AE: Adverse event
CI: Confidence interval
COVID-19: Coronavirus disease 19
EDC: Electronic Data Capture System
EMA: European Medicines Agency
EPSS: Enhanced passive safety surveillance
HCP: Healthcare professional
IIV4: Quadrivalent split-virion inactivated influenza vaccine
NH: Northern hemisphere
PRAC: Pharmacovigilance Risk Assessment Committee
PV: Pharmacovigilance
SmPC: Summary of Product Characteristics
VC: Vaccination card
WHO: World Health Organization
