# Supplementary material for: Enhanced passive safety surveillance of a quadrivalent inactivated split virion influenza vaccine in Finland during the influenza season 2020/21
Source: BMC Public Health. 2022 Aug 8;22:1506. doi: 10.1186/s12889-022-13898-z (PMC9358077; doi:10.1186/s12889-022-13898-z)
Supplement: Supplementary file 1 — Additional file 1: Table S1. Vaccinee reporting rate by PRAC adverse drug reactions occurring within 7 days after vaccination. [file 12889_2022_13898_MOESM1_ESM.docx]

**Table S1**. Vaccinee reporting rate by PRAC adverse drug reactions occurring within 7 days after vaccination

| **PRAC ADRs of interest** | | Total number of VCs distributed N= 1008 | |
| --- | --- | --- | --- |
|  |  | **n** | **(%) (95% CI)** |
| **PRAC ADRs of interest** | | 72 | 7.14 (5.63, 8.91) |
| **Injection site reactions** | | 45 | 2.18 (1.37, 3.29) |
| Vaccination site pain | 19 | 1.88 (1.14, 2.93) |  |
| Vaccination site erythema | 15 | 1.49 (0.84, 2.44) |  |
| Vaccination site swelling | 11 | 1.09 (0.55, 1.94) |  |
| **Myalgia** | | 9 | 0.89 (0.41, 1.69 |
| **Headache** | | 7 | 0.69 (0.28, 1.43) |
| **Fever** | | 3 | 0.30 (0.06, 0.87) |
| **Nausea** | | 3 | 0.30 (0.06, 0.87) |
| **Arthralgia** | | 2 | 0.20 (0.02, 0.71) |
| **Malaise** | | 2 | 0.20 (0.02, 0.71) |
| **Allergic and hypersensitivity reactions** | | 1 | 0.10 (0.00, 0.55) |

VCs: Vaccination card
